# The magnitude of perinatal depression and associated factors among women in Kutaber woreda public health institution and Boru Meda general hospital, Ethiopia, 2022: a cross-sectional study

**DOI:** 10.3389/fpsyt.2023.1302168

**Published:** 2024-01-22

**Authors:** Jemal Seid, Emam Mohammed, Nigusie Cherie, Husnia Yasin, Elsabeth Addisu

**Affiliations:** ^1^Department of Psychiatry, College of Medicine and Health Sciences, Wollo University, Dessie, Ethiopia; ^2^School of Public Health, College of Medicine and Health Sciences, Wollo University, Dessie, Ethiopia

**Keywords:** perinatal mental disorder, antenatal depression, postnatal depression, perinatal care, pregnancy, Ethiopia

## Abstract

**Background:**

Perinatal depression, characterized by the presence of depressive symptoms during pregnancy and/or within the first 12 months postpartum, poses a significant global public health concern. It contributes to a multitude of health risks for mothers, their infants, and their families. Understanding of perinatal depression and its associated factors is crucial for effective prevention and intervention strategies. However, there is a lack of comprehensive research on this topic in Ethiopia. Therefore, this study aims to determine the prevalence and factors contributing to perinatal depression among Ethiopian women.

**Methods:**

An institutional-based cross-sectional study was conducted, involving 552 women receiving perinatal services at Kutaber district health institution and Boru Meda General Hospital. Study participants were selected through systematic random sampling techniques. Perinatal depression was assessed using the Depression, Anxiety, and Stress Scale-21 (DASS-21). The associations between various determinants and perinatal depression were examined using binary logistic regression, and factors with a *p*-value of less than 0.2 were included in the multiple logistic regression analysis. A *p*-value less than 0.05 was considered statistically significant.

**Results:**

The prevalence of perinatal depression was found to be 32.2%. The prevalence of perinatal depression was found to be 32.2%. Factors significantly associated with perinatal depression included being a student [adjusted odds ratio (AOR) = 4.364, 95% confidence interval (CI): 1.386, 13.744], experiencing excessive pregnancy-related concerns (AOR = 1.886, 95% CI: 1.176, 3.041), past substance use (AOR = 2.203, 95% CI: 1.149, 4.225), the presence of anxiety symptoms (AOR = 3.671, 95% CI: 2.122, 6.352), experiencing stress symptoms (AOR = 6.397, 95% CI: 3.394–12.055), and daytime sleepiness (AOR = 2.593, 95% CI: 1.558, 4.316).

**Conclusion:**

The findings of this study indicate a relatively high prevalence and valuable factors associated with perinatal depression. It highlights the need for a comprehensive approach to perinatal mental health that takes into account not only the biological aspects of pregnancy but also the psychological, social, and lifestyle factors that can impact a person’s mental well-being during this critical period.

## Introduction

Perinatal depression, a prevalent mental health issue, affects pregnant individuals and new mothers during the perinatal period, encompassing pregnancy and the postpartum period (1, 2). While extensively studied in developed countries, perinatal depression remains a significant concern in developing countries, where healthcare resources may be limited (3–17). Perinatal depression is characterized by the occurrence depressive symptoms during pregnancy and/or within 12 months following delivery (15–18). Globally, perinatal depression is a major public health concern due to its direct association with altered mother-to-child interaction, diminished outcomes in child development, and considerable personal, economic, as well as social costs (15, 19, 20). It is the most common mental health problems experienced by a woman during perinatal period (3, 4, 10–14) and is linked with increased risks of maternal & infant mortality and morbidity (5–9).

Due to physiological, psychological, hormonal, and social changes during pregnancy and the postpartum period, the likelihood of experiencing emotional disturbances such as depression may increase (21–23). Approximately 70% to 80% of all new mothers experience some negative psychological feelings or mood swings during pregnancy, delivery, and the first year after giving birth (24). Becoming a mother is a significant life transition that impacts a woman’s sense of self and identity. It involves navigating various emotional, physical, and social changes. These changes often require women to reassess their roles, goals, and aspirations both within and outside of motherhood. This process of identity development is complex and can contribute to a mother’s depression (25, 26). In the perinatal period, parents acquire new roles, responsibilities, and knowledge and respond to changes in personal identity, relationships, and family dynamics (27–31).

Depressive symptoms during the perinatal period may have devastating consequences not only for maternal health but also for mother-to-child interaction and their child’s biological, physiological, social, and cognitive development (5, 7, 27, 32, 33).

Identity development before becoming a parent plays a crucial role in one’s ability to cope with the responsibilities and challenges of parenthood. Developing a solid adult identity allows individuals to have a strong sense of self, a clear understanding of their values, and a firm grasp on their personal goals and aspirations (34). Transitioning to parenthood can be overwhelming in itself, and without a strong sense of self, individuals may find it challenging to navigate the changes and adjustments that come with this new role. They may feel a loss of personal freedom and struggle to find a balance between their own identity and the responsibilities of being a parent. When individuals enter parenthood without a well-established identity, they may face various challenges. Firstly, they may struggle with a lack of self-confidence and uncertainty about their abilities as a parent. Secondly, they may experience difficulties in setting boundaries and establishing a sense of self within the parental role. They may struggle with balancing their own needs and desires with the demands of their child, leading to feelings of frustration, resentment, or guilt (35).

Regarding perinatal depression, neurobiological birth dynamics play a pivotal role. The perinatal period is characterized by significant hormonal fluctuations, especially during pregnancy and after childbirth, which can influence maternal mental health. Additionally, the neurobiological changes associated with pregnancy and childbirth can impact the mother’s stress response and emotional regulation systems, contributing to the risk of perinatal mood disorders such as depression and anxiety (36–38).

In developing countries maternal mental health problems especially depression during perinatal period become the most challenging issue (39–41). According to WHO reports depression is the fourth leading cause of disability worldwide (42). Depression is also predicted to become the leading cause of disease burden by 2030, and it is already the leading cause of disease burden in women worldwide (42, 43). Perinatal depression rates vary globally, with studies reporting different prevalence rates. In the USA, rates ranged from 18.4% to 40.4% among women (44). In rural China, 13% exhibited symptoms of depression (14) while in Italy, 18.7% of perinatal caregiver professionals reported depression symptoms (45, 46). Low- and middle-income countries showed a wide range of 3%–50% prevalence (47). In Malaysia, depression rates during the perinatal period varied from 1.9% to 82.1% in developing countries and 5.2% to 74.0% in developed countries (48).

During the COVID-19 outbreak in Mexico, 39.2% of women developed depression symptoms (30), and in Portugal, 13%–16% experienced postnatal depression (32). Among Australian fathers, 10% experienced perinatal depression (49), and in Brazil, the prevalence was 24.3% during pregnancy and 10.8% postpartum (50). In Egypt, 40% of university students exhibited depressive symptoms (51). and in Ghana, 9.9% experienced antenatal depression (52). Various studies among pregnant women reported rates ranging from 24.94% to 58% for depressive symptoms (53–58). Several factors were associated with perinatal depression across these studies. Unemployment (45) and financial problems (13) were common determinants. Low social support, single status, lower education, unemployment, financial instability, and older age increased the risk (4, 59). Other systematic review and meta-analysis study in China shows that educational level and economic status of families were significantly correlated with perinatal depression (60). The absence of a partner (50), maternal age less than 30 years old, never being married (52), and low economic status and low economic status (50, 52) were also significant factors with perinatal depression.

Clinically, a history of depression, physical trauma (61), lack of physical activity (62), poor physical health (63), sleep disturbance (27), poor health status, and history of traumatic experiences (64), physical & sexual abuse (28), domestic violence (60), poor relationships with their parent (7), family conflict, lack of decision-making power and poor social support (14) were linked to perinatal depression. Lifetime stressful event exposure (7, 64, 65) perinatal smoking and/or the use of alcohol (50, 60, 66), and husband smoking status (57) were additional contributing factors.

Obstetric factors such as multiparity (50, 60), unintended pregnancy (52, 54, 55), and previous pregnancy loss (52, 67) having more than 4 living children (7, 57, 68), emotional detachment during childbirth (69), history of lifetime abortion (54), age at marriage (57), obstetric complications in previous and/or this pregnancy (57, 58), violence during pregnancy (70, 71) were also factors associated with perinatal depression. Generally, perinatal depression in developing countries particularly in Ethiopia poses a significant public health challenge, with potentially severe consequences for maternal and child well-being. Understanding the prevalence and associated factors in developing countries is crucial for effective prevention and intervention strategies. However, there is a lack of comprehensive research addressing the prevalence and the factors contributing to perinatal depression in Ethiopia. So, this study aimed to determine the prevalence and its associated factors of perinatal depression among women in Ethiopia.

## Methods and materials

### Study areas, design, and period

An Institution based cross-sectional quantitative study design was conducted to assess the prevalence and associated factors of perinatal depression among women attending perinatal services in Kutaber district public health facilities and Boru Meda general hospital from January to August 2022.

### Population, sample size and sampling procedure

All women who attend antenatal care, postnatal care & child vaccination program in the last 12 months after delivery in the selected public health facilities of the district and Boru Meda general hospital. Women who attended perinatal health services in the selected public health of Kutaber district and Boru Meda General Hospital during the study period. Inclusion criteria for the enrolling women were the age of ≥18 years, having regular ANC & PNC follow up as well as women who came for delivery during the study period if they are volunteers. Women who are unable to communicate, severely ill and previous diagnosis of psychiatric disorder were excluded from the study. To study the associated risk factors with perinatal depression Epi. Info. Version 7, for double population proportion, was used with an assumption of; a two-sided confidence level (1-alpha) (95%), power (chance of detecting) (80%), the ratio of controls to cases (1), the hypothetical proportion of controls with exposure, and hypothetical proportion of cases with exposure from previous research findings of related works (54, 57). Accordingly, the researcher gets the largest study participant in the second objectives sample size calculation which is 502 and takes it by adding 10% non-response rate. Finally, the population was also proportionally allocated. The study respondents were recruited using a systematic random sampling method. The total estimated number of women that visited the five public health centers and the one general hospital per day is 65 patients. Since the number of required test subjects were 552, a sampling interval of three were used as the constant difference between subjects. The first starting number of each study site was selected randomly using the lottery method from the registration counter. A structured interviewer-administered questionnaire was used to obtain socio-demographic and relevant associated information from the respondents.

### Data collection tools and procedure

Perinatal depression was measured using the depression content of Depression Anxiety Stress Scale-21 (DASS-21). DASS-21 has been widely used in studying perinatal psychological health (72–76). Each item in DASS-21 is rated using a 4-point scale (0 for always false or not applicable to 3 for always true or totally applicable). Higher scores indicate greater distress levels. The internal consistency reliability of DASS-21 was very impressive in Ethiopia with Cronbach’s alpha 0.75, 0.72, 0.86, and 0.95 for DASS depression, anxiety, stress, and total scales, respectively (77). These coefficients demonstrated good internal consistencies. The cut-off values for DASS depression, anxiety and stress were 9, 7, and 14, respectively (74, 75). Based on the will of the women those who had scores for depression, anxiety, and stress scale higher than the cut point were linked to the psychiatric ward for further diagnostic investigation.

The Epworth Sleepiness Scale was used as a subjective measure of a woman day time sleepiness. The test had a list of eight situations in which women were rated to become sleepy on a scale of 0, no chance of dozing, to 3, high chance of dozing (78, 79). The total score was based on a scale of 0 to 24. The scale estimates whether women were experiencing excessive sleepiness that possibly requires medical attention (78–82). Socio-demographic characteristics and obstetric variables: trimester, having previous pregnancy, previous pregnancy & labor complication, previous history of stillbirth, previous history of abortion, plan current pregnancy, previous ANC follow-up, current pregnancy complication and previous psychiatric history was collected by a structured and pre-tested questionnaire.

The Baby’s father’s support, partner’s feeling on current pregnancy, community support, and substance use history was also collected by a structured questionnaire.

### Operational definition

#### Perinatal period

The period starting from pregnancy (antenatal) until the end of the first year after a baby is born (postnatal) (6, 57, 83, 84).

#### Anxiety symptoms

Scoring above the cut-off scores (7) on DASS-21 scales used for assessing perinatal anxiety symptoms (11).

#### Stress symptoms

Scoring above the cut-off scores (14) on DASS-21 scales used for assessing perinatal stress symptoms (11).

#### Daytime sleepiness

Those women having ESS score of >10 (85–91).

### Data quality assurance

To assure the data quality high emphasis was given in designing data collection instruments. The questionnaires were pretested Haroye health post on 10% of the sample size to check consistency and length of time each questioner took, sampling method and techniques.

Training was provided for data collectors, and supervisors and before data collection the questioners were checked its simplicity, clarity and understandability. Checking and re-checking of the data was employed to identify whether the data was completely filled or not by double data entry. Daily supervision of the data collection process was implemented. One day of training was given for data collectors and supervisors. To ensure the quality of data, a properly designed standardized data collection tool was used.

### Data analysis procedure

Each completed questionnaire was coded. The data was checked and cleaned by entering into Epi data version 4.6 and was exported into Statistical Package for the Social Sciences SPSS window version 26, for analysis. Descriptive statistics were employed to estimate the prevalence of perinatal depression. Bivariate analyses (binary logistic regression) were carried out between the predictors and outcome variables.

Using significant variables (*p* < 0.2) from binary logistic regression models, a multivariable logistic regression model was fitted to identify the independent predictors of perinatal depression. The strength of association was measured by odds ratios with 95% confidence intervals. Statistical significance was declared at *p* < 0.05. To measure the fitness of the data to the model Hosmer–Lemeshow test was conducted. The small *p*-value <0.05 of the goodness of fit test means that the model is not good fit. And also apply to the binary logistics regression assumptions were conducted that were linearity of the independent variable, the inclusion of relevant variable, meaningful coding, independent observation, no multicollinearity and sufficiently large sample size.

### Ethical consideration

The studies involving human participants were reviewed and approved by Ethical Committee of Wollo University College of Medicine and Health Science with an ethical review number (RCSPG-191/14). All procedures performed were in accordance with the ethical standards of the institutional and national research committee at which the studies were conducted and with the 1964 Helsinki Declaration and its later amendments or comparable ethical standards. A written informed consent was obtained from each participant or their parents &/or legal guardians.

## Results

### Sociodemographic and husband-related characteristics of the study subject

A total of 552 women were recruited for the study, and all of them completed the questionnaire, resulting in a 100% response rate. The respondents were all aged between 18 and 45 years, with a mean age of 29.6 ± 5.385, a median of 29, and a mode of 30. Geographically, 295 (53.4%) lived in rural areas. The majority of the participants, 532 (96.4%), were of Amhara ethnicity, 363 (56.8%) were Muslim, 209 (37.9%) had completed primary school, 443 (80.3%) were married, 321 (58.2%) reported not knowing their monthly income, and 245 (44.4%) had medium levels of social support. Of the total 552 respondents, 333 (60.3%) had employed husbands (Table 1).

**Table 1:** Socio-demographic characteristics of study subject (*n* = 552).

	Variables	Frequency	Percentage
Age	<24	99	17.9
	24–35	392	71.0
	>35	61	11.1
Residency	Rural	295	53.4
	Urban	257	46.6
Ethnicity	Amhara	532	96.4
	Tigray	14	2.5
	Afar	4	0.7
	Oromo	2	0.4
Religion	Muslim	363	65.8
	Orthodox	172	31.2
	Catholic	6	1.1
	Protestant	9	1.6
	Others	2	0.4
Educational status	Unable to read and write	133	24.1
	Primary school	209	37.9
	Secondary school	115	20.8
	Diploma & above	95	17.2
Marital status	Single	64	11.6
	Married	443	80.3
	Divorced	28	5.1
	Widowed	17	3.1
Employment	Student	41	7.4
	Housewife	335	60.7
	Merchant	51	9.2
	Government	93	16.8
	Private	19	3.4
	Others	13	2.4
Monthly income	Do not know	321	58.2
	Up to 1,000	44	8.0
	>1,001	187	33.9
Husband employment status	Employed	364	65.9
	Not employed	183	33.2
	Has not husband	5	0.9
Marital conflict	Absent	231	41.8
	Present	316	57.2
	Has not husband	5	0.9
Support from her husband	Enough	437	79.2
	Not enough	110	19.9
	Has not husband	5	0.9

### Description of respondents by obstetric & other psychosocial related factors

Out of the total respondents, 459 (83.2%) reported having happy feelings during pregnancy, 443 (80.3%) experienced pregnancy after marriage, 341 (61.8%) had 1–3 children, 391 (70.8%) had a history of normal vaginal delivery, and 485 (87.9%) had no history of sexual violence. Additionally, 199 (36.1%) had a history of substance use, and 153 (27.7%) were living with other chronic medical illnesses (Table 2).

**Table 2:** Obstetric and other psychosocial characteristics of study subject (*n* = 552).

	Variables	Frequency	Percentage
Feeling on current pregnancy	Unhappy	93	16.8
	Happy	459	83.2
Is the current pregnancy planned?	Unplanned	119	21.6
	Planned	433	78.4
Premarital pregnancy	No	443	80.3
	Yes	109	19.7
Parity	Nulliparous	160	29.0
	1–4 previous live birth	334	60.5
	>previous live birth	58	10.5
Excessive pregnancy-related concern	Present	189	34.2
	Absent	363	65.8
Past history of obstetric complication	Present	204	37.0
	Absent	348	63.0
Bad attitude towards current pregnancy	Yes	115	20.8
	No	437	79.2
Number of children	No child	136	24.6
	1–3 Children	341	61.8
	4 and above children	75	13.6
Delivery outcome (last birth)	Stillbirth	88	15.9
	Live birth	423	76.6
	Still pregnant	41	7.4
Mode of delivery (last delivery)	Cs	108	19.6
	Other	12	2.2
	Still first pregnant	41	7.4
	Normal vaginal delivery	391	70.8
The presence of adverse life events?	No	399	72.3
	Yes	153	27.7
Illness in the family member?	Absent	434	78.6
	Present	118	21.4
Financial conflict?	Absent	310	56.2
	Present	242	43.8
History of domestic violence?	No	495	89.7
	Yes	57	10.3
History of sexual abuse?	No	485	87.9
	Yes	67	12.1
History of physical abuse?	No	484	87.7
	Yes	68	12.3

### Perinatal depression scores of the participants

**Figure 1:**
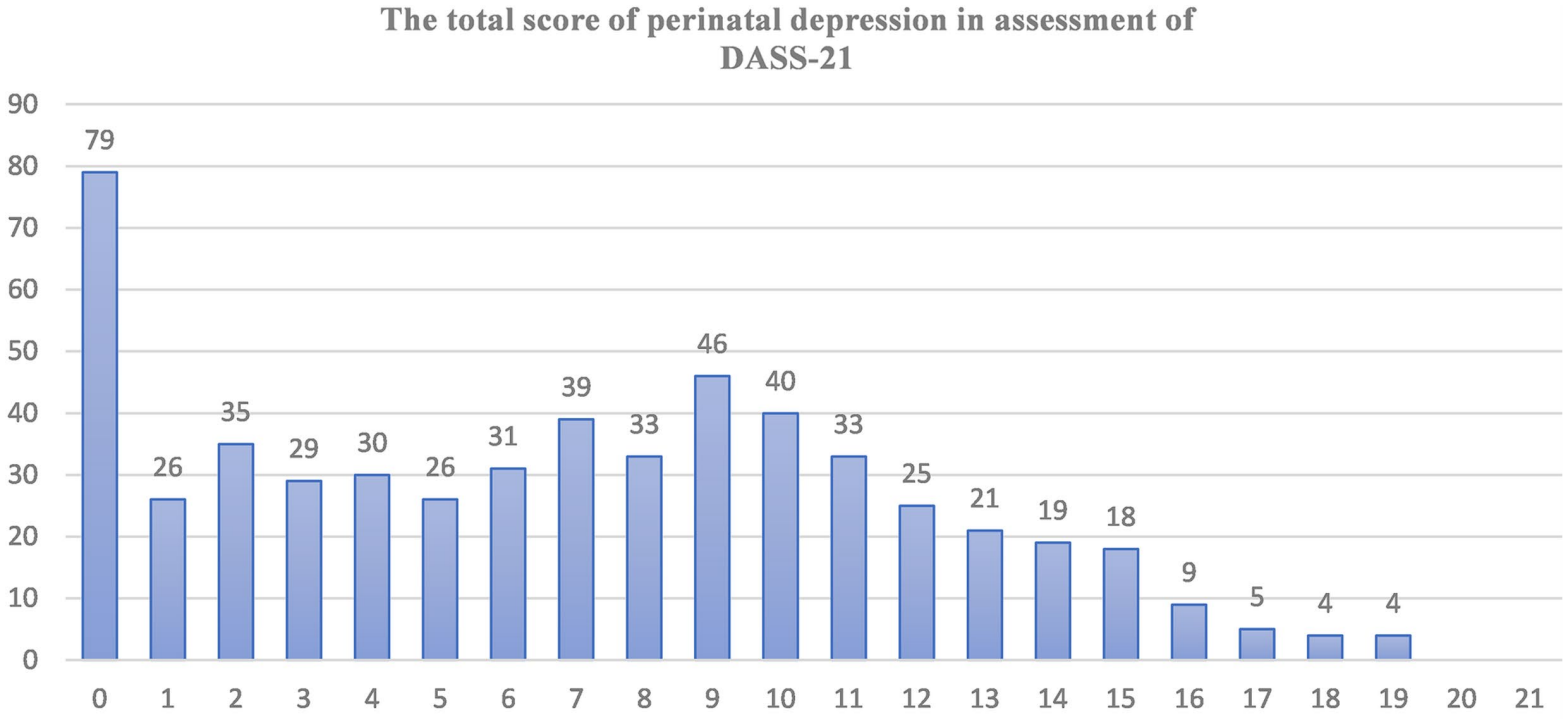
Graph of the total response of depression symptoms (from 0 up to 21) during perinatal period on the seven depression related items of DASS-21 scales. The *X*-axis indicates the sum scores (0–21) of depressions related symptoms assessment. *Y*-axis indicates the number of participants who score different level of depression related symptoms.

The overall prevalence of perinatal depression was 32.2% (95% CI, 27.87–36.62). The cutoff point for depression on the DASS-21 scale was set at 9, and accordingly, 178 (32.2%) of study subjects met the criteria for depression symptoms. The depression scores on the DASS-21 scale ranged from 0 to 19, with a median score of 7 (Figure 1).

### Distribution of perinatal depression among sociodemographic factors of the respondent

Among the respondents who developed perinatal depression, 125 (70.2%) were in the age group of 24–35 years, 92 (51.7%) lived in rural areas, 132 (74.2%) were married, 120 (67.4%) were employed, 94 (52.8%) did not have a known monthly income, and 85 (47.8%) reported having a moderate level of social support. Among the respondents who developed depression, 136 (76.4%) reported happy feelings during pregnancy, 133 (74.7%) reported that pregnancy was planned and wanted, 134 (80.3%) reported pregnancy occurring after marriage, 97 (54.4%) had a history of past obstetric pregnancy complications, 106 (59.6%) had 1–3 children, and 90 (50.6%) experienced adverse life events. Socially, 100 (56.2%) were exposed to financial conflicts. Clinically, among the women who developed depressive symptoms, 95 (53.4%) and 86 (48.3%) reported substance use in their lifetimes and in the past 3 months, respectively.

### Factors associated with perinatal depression

Bivariate analyses were conducted between perinatal depression and independent variables (sociodemographic, obstetric and clinical factors). Finally, all individual factors with a *p*-value <0.20 in bivariate logistic regression analysis were entered into multivariate logistic regression for further analysis. According to the multivariate analysis, being a student (AOR = 4.364, 95% CI: 1.386, 13.744), excessive pregnancy-related concern (AOR = 1.886, 95% CI: 1.176, 3.041), ever substance use (AOR = 2.203, 95% CI: 1.149, 4.225), the presence of anxiety symptoms (AOR = 3.671, 95% CI: 2.122, 6.352), stress symptoms (AOR = 6.397, 95% CI: 3.394–12.055), and daytime sleepiness (AOR = 2.593, 95% CI: 1.558, 4.316) were significantly associated with perinatal depression (Table 3).

**Table 3:** Factors associated with perinatal depression (*n* = 552) (bivariate & multivariate logistic regressions).

Variable	Depression			Bivariate analysis			Multivariate analysis		
	*N*_Total_	Yes	No	*p*-value	COR	95% CI	*p*-value	AOR	95% CI
**Educational status**									
Unable to read and write	133	36	97	0.569	0.845	0.473–1.509	0.242		
Primary school	209	79	130	**0.220** ^*^	1.383	0.823–2.323	0.703		
Secondary school	115	34	81	0.880	0.955	0.528–1.728	0.607		
Diploma & above	95	29	66	1					
**Marital status**									
Married	443	132	311	1					
Single	64	23	41	**0.094** ^*^	0.393	0.132–1.171	0.493		
Divorced	28	13	15	0.421	0.607	0.179–2.052	0.649		
Widowed	17	10	7	**0.016** ^*^	0.297	0.111–0.797	0.361		
**Employment**									
Government	93	30	63	1					
Student	41	7	34	**0.115** ^*^	0.329	0.083–1.312	**0.012** ^**^	**4.364**	**1.386–13.744**
Housewife	335	120	215	0.846	0.893	0.286–2.791	0.412		
Merchant	51	15	36	0.531	0.667	0.187–2.372	0.274		
Private	19	1	18	**0.039** ^*^	0.089	0.009–0.889	0.358		
Others	13	5	8	0.657	0.762	0.230–2.527	0.210		
**Monthly income**									
Don’t know	321	94	227	**0.127** ^*^	0.742	0.505–1.088	0.118		
Up to 1000	44	17	27	0.728	1.128	0.573–2.218	0.203		
>1,001	187	67	120	1					
**Current pregnancy**									
Unplanned	119	45	74	**0.143** ^*^	1.372	0.899–2.094	0.726		
Planned	433	133	300	1					
**Excessive pregnancy-related concern**									
Present	189	76	113	**0.004** ^*^	1.721	1.189–2.492	**0.009** ^**^	**1.886**	**1.170–3.041**
Absent	363	102	261	1					
**Past history of obstetric complication**									
Present	204	81	123	1					
Absent	348	97	251	**0.004** ^*^	1.704	1.182–2.456	0.280		
**Bad attitude towards current pregnancy**									
Yes	115	44	71	**0.122** ^*^	1.401	0.914–2.149	0.879		
No	437	134	303	1					
**Number of children**									
No child	136	36	100	1					
1–3 children	341	106	235	0.320	1.253	0.803–1.955	0.238		
4 and above children	75	36	39	**0.002** ^*^	2.564	1.419–4.634	0.494		
**The presence of adverse life events?**									
No	399	106	293	1					
Yes	153	72	81	**0.000** ^*^	2.457	1.668–3.620	0.119		
**Illness in the family member**									
Absent	434	120	314	1					
Present	118	58	60	**0.000** ^*^	2.529	1.666–3.841	0.214		
**Financial conflict**									
Absent	310	78	232	1					
Present	242	100	142	**0.000** ^*^	2.095	1.458–3.009	0.791		
**History of domestic violence?**									
No	495	147	349	1					
Yes	57	31	25	**0.000** ^*^	3.319	1.895–5.812	0.998		
**History of sexual abuse?**									
No	485	145	350	1					
Yes	67	33	24	**0.000** ^*^	3.467	2.053–5.854	0.115		
**Substances use in lifetime**									
No	353	83	270	1					
Yes	199	95	104	**0.000** ^*^	2.972	2.050–4.308	**0.017** ^**^	**2.203**	**1.149–4.225**
**Any substances in the past three months?**									
No	366	92	274	1					
Yes	186	86	100	**0.000** ^*^	2.561	1.765–3.718	0.455		
**Anxiety**									
Have no anxiety	313	46	267	**1**					
Have anxiety	239	132	107	**0.000** ^*^	7.161	4.783–10.721	**0.000** ^**^	**3.671**	**2.122–6.352**
**Stress symptoms**									
Have no stress	439	94	345	**1**					
Have stress	113	84	29	**0.000** ^*^	10.631	6.580–17.177	**0.000** ^**^	**6.397**	**3.394–12.055**
**Sleep problems**									
Haven’t day time over sleep	434	115	319	1					
Have day time oversleep	118	63	55	**0.000** ^*^	3.177	2.088–4.835	**0.000** ^**^	**2.593**	**1.558–4.316**
**Chronic medical illness?**									
No	399	104	295	1					
Yes	153	74	79	**0.000** ^*^	2.657	1.803–3.916	0.347		

^*^p-value < 0.20, ^**^Statically significant at *p* < 0.05 and *p*-value of Hosmer and Lemeshow goodness of fit test was = **0.616**.

## Discussion

The prevalence of perinatal depression in this study was 32.2%, which was consistent with findings from studies conducted in Nepal 36% (92), Brazil 25.9% (67), Latin America 28.2% (93), Vietnam 29.9% (94), South Africa 39% (71) Egypt 40% (51) and Ethiopia 35.9% (54). However, it was lower than the prevalence reported in studies conducted in the United States 40.4% (44), UK 47.3% (61), Mexico 39.2% (30), Rwanda 37.6% (7) and Ethiopia 58% (56). On the other hand, it was higher than the prevalence in studies conducted in Colombia 22.36% (9), Canada 17.9% (63), Norwegian 2% (28), China 13% (14), Malaysia 12.5% (95) Australia 19.8% (41), Asia 20% & 21.8% (96), Ghana 24.3% (50) and other parts of Ethiopia (24.9, 26.7, 25.8%) (53, 57, 58). Generally, the discrepancy of the prevalence of depression during the perinatal period might be a result of a difference in assessment tools, geographical areas, sample size, health status & cultures of the study subject and the study setting. Even though there is discrepancy, the result is supported by many other studies carried out elsewhere perinatal depression as a significant public health concern among women in the perinatal period (7, 28, 30, 44, 51, 61, 67, 70, 95, 97–99).

Being a student during the perinatal period was found to be a significant risk factor for perinatal depression (AOR = 4.364, 95% CI: 1.386, 13.744). This finding suggests that the unique challenges and stressors faced by student mothers may contribute to their vulnerability to depression. Early parenting presents numerous challenges, and these challenges are closely linked to the need for a mature and well-developed adult identity. Becoming a parent requires individuals to navigate significant changes in their roles, responsibilities, and priorities. They must also grapple with their own emotional and psychological development, as they transition from being a child or young adult to assuming the role of a caregiver (34).

These stressors might include academic demands, financial pressures, and social support deficits. Academically, student mothers may experience additional stress due to the demands of attending classes, completing assignments, and studying while also being pregnant or caring for a newborn (34). Financial pressures like balancing the costs of education, such as tuition fees, textbooks, and other expenses, with the financial responsibilities of pregnancy or caring for a child can be challenging for student mothers (100). Socially, student mothers may have limited social support networks due to being away from their families or lacking access to resources that could provide assistance during pregnancy and early motherhood (100–102) This lack of support can contribute to feelings of isolation and increased stress levels. It’s important to note that these factors are not the sole causes of perinatal depression in student mothers, but they may contribute to their increased vulnerability. The combination of hormonal changes, sleep deprivation, and the demands of parenting can be particularly challenging for individuals who have not yet developed a strong sense of identity. They may struggle with feelings of isolation, inadequacy, and a sense of losing their own identity within the role of motherhood. These factors can contribute to the development or exacerbation of perinatal depression (35).

It’s crucial for healthcare professionals, educational institutions, and support networks to be aware of these challenges and provide appropriate support and resources to help student mothers during this critical period (35).

Excessive worry or concern related to pregnancy was identified as another contributing factor (AOR = 1.886, 95% CI: 1.176, 3.041). This result indicates that heightened anxiety or preoccupation with pregnancy-related issues can increase the risk of perinatal depression. This might be due to the emotional toll of worrying about the health and well-being of both the mother and the baby. It’s important to consider the possible mechanisms behind this relationship. Pregnancy can be a time of immense change and uncertainty, and concerns about the baby’s health, the mother’s well-being, and the impending responsibilities of parenthood may contribute to increased levels of concern/worry. This, in turn, can impact a woman’s mental health during pregnancy and the postpartum period. Healthcare providers should be attuned to the psychological well-being of expectant mothers and offer appropriate support and resources to help them navigate their anxieties. Additionally, interventions focused on stress reduction, coping strategies, and emotional support may be beneficial in reducing the risk of perinatal depression among individuals experiencing heightened pregnancy-related worries.

The study found that a history of substance use is associated with an increased risk of perinatal depression (AOR = 2.203, 95% CI: 1.149, 4.225). This result was supported by other studies conducted in China (40), in South Africa (19) and any other countries (50, 60, 66). Substance use can have adverse effects on mental health and can exacerbate depressive symptoms. It may also impair decision-making and coping mechanisms, making it harder for individuals to manage their emotional well-being during perinatal period.

Similar to other studies in China (99, 103), presence of anxiety symptoms was strongly associated with perinatal depression (AOR = 3.671, 95% CI: 2.122, 6.352). Anxiety and depression often cooccur, and this finding underscores the importance of assessing and addressing both conditions during the perinatal period. Understanding the relationship between anxiety and perinatal depression is crucial for healthcare professionals and researchers to effectively screen, diagnose, and provide appropriate interventions. It is well-known that anxiety and depression often cooccur, and this comorbidity can worsen the overall mental health of pregnant and postpartum women.

High levels of anxiety can directly contribute to the development or exacerbation of depressive symptoms. Anxiety increases stress levels, which can disrupt a woman’s ability to cope with the challenges of pregnancy and early motherhood. Moreover, anxiety can interfere with sleep, appetite, and overall well-being, further exacerbating the risk of perinatal depression (99). Addressing both anxiety and depression during the perinatal period is crucial for optimal mental health outcomes. Early identification and intervention can help prevent the escalation of symptoms and improve overall maternal well-being. By recognizing and addressing the interconnected nature of these mental health conditions, healthcare providers can provide holistic care to pregnant and postpartum women, promoting better outcomes for both the mother and the child.

Consistent with the results of previous studies (104), stress symptoms were found to have a substantial impact on perinatal depression (AOR = 6.397, 95% CI: 3.394–12.055). High levels of stress, whether related to personal life, relationships, or external factors, can overwhelm pregnant or postpartum individuals, making them more susceptible to depression. Stress, whether stemming from personal life, relationships, or external factors, can be particularly challenging during the perinatal period (105). Pregnancy and early parenthood can bring about various stressors, such as financial concerns, changes in identity, lack of support, and sleep deprivation. Additionally, hormonal changes and physical discomforts associated with pregnancy can further exacerbate stress levels.

When stress becomes overwhelming and persistent, it can disrupt an individual’s emotional well-being and increase the likelihood of developing depression. The study highlights the importance of identifying and addressing stress symptoms during the perinatal period to mitigate the risk of depression. Interventions aimed at reducing stress and promoting emotional well-being during pregnancy and postpartum can be beneficial in preventing or minimizing the severity of perinatal depression (35). These interventions may include stress management techniques, social support networks, cognitive-behavioral therapy, and mindfulness-based approaches. Engaging in regular physical activity, practicing relaxation techniques, and seeking professional help when needed are also essential strategies for managing stress during this critical period.

Daytime sleepiness emerged as a significant factor associated with perinatal depression (AOR = 2.593, 95% CI: 1.558, 4.316). A similar finding was reported from the studies conducted in Sweden (106), and in USA (107). Disrupted sleep patterns are a common experience for many individuals during pregnancy and early parenthood. These disruptions can be caused by a variety of factors, including physical discomfort, hormonal changes, anxiety, and the demands of caring for a new infant. Such disruptions can lead to significant daytime sleepiness, which may in turn contribute to mood disturbances and increase the risk of perinatal depression (106). Addressing sleep issues as part of perinatal care can involve a range of strategies, including providing education about sleep hygiene, offering guidance on managing discomfort and anxiety, and in some cases, considering the appropriate use of medication under the supervision of a healthcare professional. By understanding and addressing sleep issues as part of perinatal mental health care, healthcare providers can potentially reduce the risk of perinatal depression and improve the overall well-being of individuals during this critical life stage.

### Limitation of the study

Recall and response biases might have occurred when completing the questionnaire. In addition, some of the independent variables like physical & sexual abuse, and the presence of suicidal wish was assessed by close-ended questions which may lead some patients to respond in an indecorous manner. Because of using a cross-sectional study design, we were not demonstrating any cause and effect association between the possible determinate factors and the outcome of interest.

## Conclusion

Globally perinatal depression is a public health concern. It contributes to the high burden of health risks faced by mothers, their child and their family. This study underscores the complex interplay of various factors that contribute to perinatal depression. It highlights the need for a comprehensive approach to perinatal mental health that takes into account not only the biological aspects of pregnancy but also the psychological, social, and lifestyle factors that can impact a person’s mental well-being during this critical period. Early identification, intervention, and support for individuals at risk for perinatal depression are crucial to improving maternal and child health outcomes. Additionally, public health campaigns and policies should focus on raising awareness about perinatal depression and promoting early detection and intervention to improve. Finally, further research and tailored interventions are warranted to address these associated factors effectively.

## Data availability statement

The original contributions presented in the study are included in the article/supplementary materials, further inquiries can be directed to the corresponding author.

## Ethics statement

The studies involving human participants were reviewed and approved by the Ethical Committee of Wollo University College of Medicine and Health Science with an ethical review number (RCSPG-191/14). The studies were conducted in accordance with the local legislation and institutional requirements. The participants provided their written informed consent to participate in this study.

## Author contributions

JS: Conceptualization, Data curation, Formal analysis, Funding acquisition, Investigation, Methodology, Project administration, Resources, Software, Supervision, Validation, Visualization, Writing – original draft, Writing – review & editing. EM: Conceptualization, Data curation, Formal analysis, Funding acquisition, Investigation, Methodology, Project administration, Resources, Software, Supervision, Validation, Visualization, Writing – original draft, Writing – review & editing. NC: Data curation, Formal analysis, Methodology, Project administration, Validation, Writing – review & editing, Investigation, Software. HY: Conceptualization, Data curation, Formal analysis, Investigation, Methodology, Software, Supervision, Visualization, Writing – review & editing. EA: Conceptualization, Data curation, Formal analysis, Methodology, Software, Validation, Visualization, Writing – review & editing.
